# Nuclear Expression of p-STAT3 Is Associated with Poor Prognosis in ER(−) Breast Cancer

**DOI:** 10.3390/clinpract12020020

**Published:** 2022-02-25

**Authors:** Tsuyoshi Nakagawa, Goshi Oda, Hiroshi Kawachi, Toshiaki Ishikawa, Kentaro Okamoto, Hiroyuki Uetake

**Affiliations:** 1 Department of Specialized Surgeries, Graduate School, Tokyo Medical and Dental University, Bunkyou-ku, Tokyo 113-8519, Japan; oda.srg2@tmd.ac.jp (G.O.); ishi.srg2@tmd.ac.jp (T.I.); okasrg2@tmd.ac.jp (K.O.); h-uetake.srg2@tmd.ac.jp (H.U.); 2 Department of Pathology, Tokyo Medical and Dental University, Bunkyou-ku, Tokyo 113-8519, Japan; kawapth1@gmail.com

**Keywords:** signal transducers and activators of transcription (STAT), breast cancer, estrogen receptor, triple negative type, luminal type

## Abstract

The activation of signal transducer and activator of transcription 3 (STAT3) has been reported in several types of cancer, where it acts as an oncogene. However, in breast cancer, the clinical role of STAT3 remains unclear. In the present study, the association between phosphorylated-STAT3 (p-STAT3) expression and clinicopathological/biological factors was examined in each subtype. p-STAT3 expression was examined in 135 cases of breast cancer by immunohistochemistry. p-STAT3 expression was not associated with clinicopathological/biological factors and prognosis in a complete cohort of breast cancer cases. However, in patients with estrogen receptor-negative (ER(−)) breast cancer and triple-negative breast cancer (TNBC), multivariate analysis showed that higher p-STAT3 expression was significantly associated with a short relapse-free survival (*p* = 0.029, HR 5.37, 95%CI 1.19–24.29). TNBC patients with p-STAT3 overexpression were found to have a poor prognosis (*p* = 0.029, HR 5.37, 95%CI 1.19–24.29). On the other hand, in ER(+) breast cancer, p-STAT3 overexpression was associated with a favorable prognosis (*p* = 0.034, HR 9.48, 95%CI 1.18–76.21). The present results suggested that STAT3 expression may play a different role in ER(−) and ER(+) breast cancer. In the future, the pharmacological inhibition of STAT3 expression may serve as an effective therapeutic strategy for ER(−) breast cancer, particularly TNBC.

## 1. Introduction

Signal transducers and activators of transcription (STATs) are latent cytoplasmic proteins that are activated to control gene expression through the phosphorylation of a single tyrosine when cells encounter ligands such as extracellular cytokines, growth factors and hormones [1,2,3]. Ligands bound to the Janus kinase receptor-associated tyrosine kinases are subsequently phosphorylated and activated, then STATs act as a transcription factor. STATs have an SRC-homology-2 domain, through which they contact the receptor, dimerize, become phosphorylated-STATs (p-STATs) and translocate into the nucleus. The STAT family comprises seven members: STAT1, 2, 3, 4, 5A, 5B and 6 [1]. STAT3 has been shown to play an important role in cancer progression and typically acts as an oncogene [4,5,6]. For example, STAT3 regulates the expression of vascular endothelial growth factor (VEGF) and is associated with angiogenesis and tumor progression [7].

The activation of STAT3 has been reported in several types of solid tumors, including head and neck, breast, prostate, pancreatic, kidney, lung, stomach and colon cancer [8,9,10,11,12,13,14,15,16,17,18,19,20,21,22,23,24]. High expression of STAT3 and p-STAT3 has often been found to be correlated with unfavorable pathological findings and/or a poor prognosis [9,15,16,18,19,20,21,22,23,24,25]. However, certain studies have reported that the high p-STAT3 expression was closely linked to a favorable prognosis in several cancer types, including breast and prostate cancer [12,14,17]. In these cancer types, unlike other solid tumors, sex hormone levels are closely associated with cancer progression [12,14,17]. p-STAT3 expression was previously investigated in breast cancer, and no correlation was identified between p-STAT3 and clinicopathological/biological factors [13]. Certain studies have shown that the high expression of STAT3 in breast cancer was associated with a favorable prognosis, whereas others have suggested that it was associated with a poor prognosis [12,13,14,15]. Therefore, the clinical role of STAT3 breast cancer remains unclear. Dolled-Filhart et al. [12] reported that STAT3 and p-STAT3 expression in nuclear is also associated with superior overall survival. Furthermore, certain studies have shown that STAT3 and p-STAT3 expression can predict the effect of chemotherapy in breast cancer [26,27]. STAT3 and p-STAT3 may, therefore, have multiple functions in breast cancer.

Breast cancer is divided into four subtypes based on the estrogen receptor (ER) and human epidermal growth factor receptor 2 (HER2) receptor expression [28]. Breast cancer that lacks receptors is known as triple-negative breast cancer (TNBC). TNBC is the most aggressive subtype, with a poor prognosis and no available targeted therapy options. There are no differences in the gene expression levels of STAT3 in each subtype, but p-STAT3 expression is characteristic of TNBC [29,30].

In the present study, the role of p-STAT3 expression in breast cancer was examined, and the association between clinicopathological/biological factors and p-STAT3 expression was analyzed in each subtype. As mentioned above, the expression of STAT3 and p-STAT3 may play different roles in hormone-dependent cancer. The difference between ER(−), particularly TNBC, and ER(+) breast cancer was also investigated.

## 2. Materials and Methods

### 2.1. Patients and Tissue Samples

Primary invasive breast cancer specimens were obtained from 135 female patients who underwent curative surgical resection at the Department of Breast Surgery, Tokyo Medical and Dental University (Tokyo, Japan) between October 2001 and April 2004. All patients provided written informed consent, and the study was approved by the ethics committee (Tokyo Medical and Dental University, Tokyo, Japan, clinical trial ID, M2000-831). The mean patient age was 53 years (range, 29–91 years). The patients had a median follow-up time of 200 months (range, 20–232 months). No patients exhibited distant metastasis at the time of surgery. Overall, 26 cases developed cancer recurrence and 21 patients succumbed to breast cancer. Recurrence means the appearance of distant metastasis. Axillary lymph node dissection was performed in all patients, and 52/135 patients (39%) were diagnosed with lymph node metastases. Resected tumor tissues were routinely fixed in formalin and embedded in paraffin.

### 2.2. Examination of Clinicopathological/Biological Characteristics

Following hematoxylin and eosin staining, histopathological examination was performed using the International Union Against Cancer Tumor-Node-Metastasis classification criteria [31]. Blood and lymphatic vessel invasion was also evaluated, and histopathological grading was based on the Elston scale [32]. Biological characteristics, including ER and HER2 expression, were evaluated by immunohistochemistry (IHC). The status of each tumor with regard to ER expression was determined by calculating the percentage of all cancer cells within a given tumor by positive nuclear staining; the cut-off value was set at 10%. HER2 status was scored using the HER2 expression criteria [33]. For primary tumors with a HER2 score of 2+, the IHC results were further validated by fluorescence in situ hybridization.

### 2.3. p-STAT3 Immunohistochemical Staining

**Figure 1:**
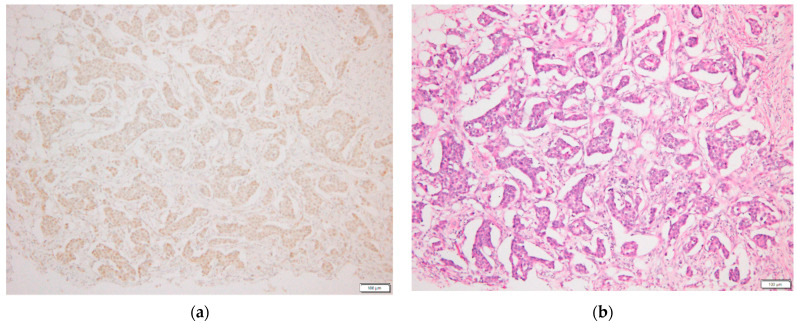
p-STAT3 positive example by immunostaining is shown. (**a**) This is a p-STAT3 positive case; (**b**) HE-stained image of the same case.

Formalin-fixed, paraffin-embedded tissues were cut into 3 µm sections, and then deparaffinized and rehydrated. Following autoclaving to maximize antigen retrieval, endogenous peroxidase activity was blocked in 0.3% hydrogen peroxide in absolute methanol for 30 min. Non-specific reactivity was blocked using a solution containing 10% normal goat serum and 10% stock blocking solution. An anti-p-STAT3 (Tyr705) goat polyclonal primary antibody (cat. no. 9131; dilution, 1:50; Cell Signaling Technology, Inc.) was used for immunostaining for 24 h at 4 °C. The slides were then incubated with Histofine^®^ Simple Stain MAX PO (cat. no. 414161; dilution, 1:1000; Nichirei Bioscience) as the secondary antibody for 30 min, according to the manufacturer’s instructions. Finally, sections were incubated in 3,3′-diaminobenzidine tetrahydrochloride and then counterstained with hematoxylin. As a negative control, non-immune goat IgG (cat. no. sc-2489; dilution, 1:100; Santa Cruz Biotechnology, Inc., Dallas, TX, USA) was used as a substitute for the primary antibody. Nuclear staining intensity was graded using the following scale: −, no staining; +, weak staining; ++, moderate staining; and +++, strong staining. Cases in which >10% of tumor cells stained ++ or +++ were considered as positive [13]. Figure 1 is a p-STAT3 positive case.

### 2.4. Statistical Analysis

Statistical analysis of STAT3 expression and clinicopathological/biological factors was carried out using Easy R (EZR; Saitama Medical Center, Jichi Medical University, Saitama, Japan), a graphical user interface for R (The R Foundation for Statistical Computing) [34]. Specifically, EZR is a modified version of R commander designed to add statistical functions frequently used in biostatistics. To estimate the significance of differences between groups, the χ^2^ and Fisher’s exact test were used, as appropriate. Survival curves were estimated using the Kaplan–Meier method, and curves were compared using the log-rank test. Survival times were determined from the date of surgery. Prognostic factors were examined by univariate and multivariate analyses using the Cox proportional hazards model. *p* < 0.05 was considered to indicate a statistically significant difference. For the RFS survival curve, Bonferroni adjustment was applied, and *p* < 0.025 was considered significant.

## 3. Results

### 3.1. Correlation between Clinicopathological/Biological Factors including p-STAT3 and Prognosis

Patient characteristics are presented in Table 1. p-STAT3 expression was not associated with clinicopathological/biological factors in any of the 135 cases. In the ER(−) group, recurrence is more common in the p-STAT3 expression positive group, while in the ER(+) group, recurrence is more common in the p-STAT3 expression negative group. Furthermore, in the TNBC group, recurrence was more common in the p-STAT3 positive group (Table 2).

### 3.2. Correlation between p-STAT3 Expression and Pathological Factors

In all cases, p-STAT3 expression was not associated with pathological factors. The same was true for ER(−), ER(+), and TNBC groups. Ten-year RFS rates are shown (Table 3).

### 3.3. Correlations between p-STAT3 Expression and Clinicopathological/Biological Factors Affecting Relapse-Free Survival (RFS)

**Figure 2:**
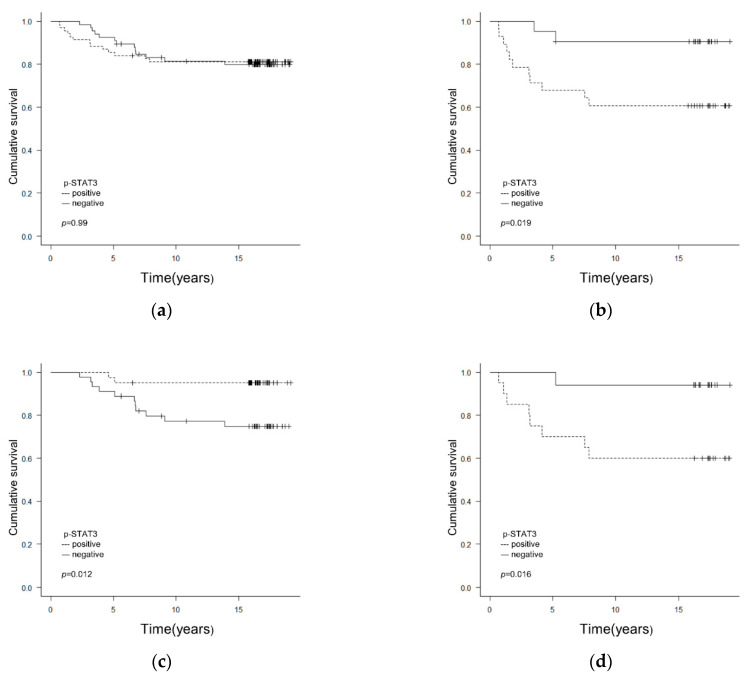
(**a**) Correlations between p-STAT3 expression and clinicopathological/biological factors affecting RFS in all cases. p-STAT3 expression was not linked to RFS in any of the 135 cases (*p* = 0.99). (**b**) Correlations between p-STAT3 expression and clinicopathological/biological factors affecting RFS in ER(−) cases. In ER(−) cases (n = 49), the RFS rate was lower in the p-STAT3(+) than in the p-STAT3(−) group (*p* = 0.019). (**c**) Correlations between p-STAT3 expression and clinicopathological/biological factors affecting RFS in ER(+) cases. In ER(+) cases (n = 86), the RFS rate was higher in the p-STAT3(−) than in the p-STAT3(+) group (*p* = 0.012). (**d**) Correlations between p-STAT3 expression and clinicopathological/biological factors affecting RFS in TNBC cases (n = 37). The RFS rate was lower in the p-STAT3(+) than in the p-STAT3(−) group (*p* = 0.016). In the RFS curves in (**b**–**d**), *p* < 0.025 was considered significant. p-STAT3, phosphorylated-STAT3; RFS, relapse-free survival; ER, estrogen receptor; TNBC, triple-negative breast cancer.

p-STAT3 expression was not associated with RFS in all 135 cases (Figure 2a). However, in ER(−) cases, the RFS rate was lower in the p-STAT3(+) group compared with that in the p-STAT3(−) group (Figure 2b). Multivariate analysis showed that tumor size and p-STAT3 expression were associated with RFS, whereas positive p-STAT3 expression was significantly associated with a shorter RFS for ER(−) patients (Table 4).

On the other hand, in ER(+) cases, the RFS rate was lower in the p-STAT3(−) compared with that in the p-STAT3(+) group (Figure 2c). Univariate analysis showed that lymph node metastasis, lymphatic vessel invasion, blood vessel invasion and p-STAT3 expression were associated with RFS (Table 4). On multivariate analysis, p-STAT3(+) expression was found to be associated with a longer RFS for ER(+) patients (Table 4).

In TNBC cases, the RFS rate was lower in the p-STAT3(+) compared with that in the p-STAT3(−) group (Figure 2d). Univariate analysis showed that tumor size and p-STAT3 expression were associated with RFS, whereas multivariate analysis showed that p-STAT3(+) expression was significantly associated with a shorter RFS in patients with TNBC (Table 4).

## 4. Discussion

In the present study, which included 135 consecutive cases, no correlations of p-STAT3 expression with clinicopathological/biological characteristics and survival were identified. However, a high p-STAT3 expression was significantly associated with a poor prognosis in ER(−) breast cancer, particularly TNBC. Therefore, in ER(−) breast cancer, p-STAT3 may be a useful poor prognostic factor.

p-STAT3 overexpression is often associated with a poor prognosis in several solid tumors, the progression of which is not linked to sex hormones, such as gastric, colorectal, lung and renal cancers [19,20,21,22,23,24]. On the other hand, studies on breast and prostate cancer have reported that a high p-STAT3 expression was associated with a favorable prognosis [12,14,17]. In breast cancer in particular, there are several studies on STAT3 and/or p-STAT3 expression and its function in human breast cancer specimens, each of which had different results. For a long time, STAT3 or p-STAT3 overexpression in breast cancer had been reported to be linked to good prognosis, but another study reported that it was associated with poor prognosis. Sheen-Chen et al. [25] showed that a high STAT3 expression was associated with a low overall 5-year survival rate. Chen et al. [15] showed that STAT3 and p-STAT3 overexpression was associated with poor survival and, potentially, lymph node metastasis. However, other studies have reported contradictory findings; Dolled-Filhart et al. [12] reported that STAT3 played the role of a tumor suppressor protein in breast cancer without lymph node metastasis, and the fact that STAT3 and p-STAT3 expression was associated with a favorable prognosis may simply mean that tumors that activate these pathways are less aggressive than tumors that progress even in the absence of STAT activation. Sato et al. [14] showed that the high p-STAT3 expression was an independent marker of good prognosis in low-grade breast cancer. In those studies, STAT3 and/or p-STAT3 overexpression in breast cancer was linked to a favorable prognosis.

The role of STAT3 in each breast cancer classified by biomarkers was recently clarified. In TNBC, the STAT3(+) group had a poor prognosis, according to an analysis using the Gene Expression Omnibus database [35]. On the other hand, in the luminal breast cancer group, STAT3(−) cases had a poor prognosis [36]. To the best of our knowledge, this is the first study including both types of results in consecutive breast cancer cases that had undergone surgery. These results suggested that the function of STAT3 may differ between ER(+) and ER(−) breast cancer.

p-STAT3 has been identified in 30–60% of primary breast cancer cases [37]. In addition, p-STAT3 is most often observed in TNBC, but can be found in all types of breast cancer [29,30]. Several studies have shown that STAT3 promotes cell proliferation and inhibits apoptosis in TNBC by increasing the expression of target genes, such as survivin, c-Myc, cyclin D1, B-cell lymphoma-2 and B-cell lymphoma-extra large [38]. Other STAT3 target genes contribute to invasion and angiogenesis, such as MMP, MMP-9, VEGF, vimentin and twist family BHLH transcription factor 1, which is the epithelial-to-mesenchymal transition inducer [39,40,41,42]. With regard to basal-like breast cancer, which is a type of TNBC, Tell et al. [43] showed that STAT3 signaling is associated with the expression of multiple genes in basal-like, but not luminal A or B, breast cancer, and that targets of STAT3 signaling in basal-like breast cancer are largely immunological and inflammatory mediators. McDaniel et al. [44] discovered novel gene signatures that were directly regulated by STAT3 in TNBC, and they found that STAT3 was enriched near invasion-related pathways. Therefore, STAT3 is considered as a tumor progression factor in TNBC.

Several trials of anti-STAT3 drugs for TNBC have been performed [45]. STAT3-targeting drugs for the treatment of TNBC have four mechanisms of action: they target upstream regulators of STAT3, act directly on STAT3 to inhibit its activation, inhibit STAT3 phosphorylation and block STAT3 DNA binding [45]. An anti-STAT3 drug may soon be useful in the treatment of breast cancer for TNBC treatment.

## 5. Conclusions

The role of STAT3 expression is different and its link to prognosis varies between ER(+) and ER(−) breast cancer. In ER(−) breast cancer, and TNBC in particular, the regulation of STAT3 may play an important role in breast cancer treatment.

## Figures and Tables

**Table 1:** Patient characteristics.

Characteristics	No.	(%)
Age(years)		
Median	52	
Range	30–91	
Tumor size		
1	63	(46.7)
2,3	72	(53.3)
Lymph node metastasis		
neg	83	(61.5)
pos	52	(38.5)
Nuclear grade		
1,2	109	(80.7)
3	26	(19.3)
Blood vessel invasion		
neg	119	(88.1)
pos	16	(11.9)
Lymphatic vessel invasion		
neg	104	(77.0)
pos	31	(23.0)
Estrogen receptor		
neg	49	(36.3)
pos	86	(63.7)
HER2		
neg	114	(84.4)
pos	21	(15.6)
Recurrence		
neg	109	(80.7)
pos	26	(19.3)
Survival		
alive	114	(84.4)
dead	21	(15.6)

**Table 2:** Relationship of p-STAT3 expression and clinicopathological/biological factors to disease recurrence in all cases, ER(−) cases, ER(+) cases and TNBC cases.

Variables	All Cases (n = 135)			ER(−) Cases (n = 49)			ER(+) Cases (n = 86)			TNBC (n = 37)		
	Non-Recurrence	Recurrence	*p*-Value	Non-Recurrence	Recurrence	*p*-Value	Non-Recurrence	Recurrence	*p*-Value	Non-Recurrence	Recurrence	*p*-Value
Tumor size												
1	57	6	0.009	21	1	0.003 *	36	5	0.56 *	18	1	0.008 *
2,3	52	20		15	12		37	8		10	8	
Lymph node metastasis												
neg	75	8	<0.001	24	5	0.104 *	51	3	<0.001 *	19	4	0.26 *
pos	34	18		12	8		22	10		9	5	
Nuclear grade												
1,2	88	21	1 *	23	9	1 *	65	12	1 *	17	6	1 *
3	21	5		13	4		8	1		11	3	
Blood vessel invasion												
neg	99	20	0.084	33	12	1 *	66	6	0.016 *	26	8	1 *
pos	10	6		3	1		7	5		2	1	
Lymphatic vessel invasion												
neg	91	13	0.001	27	7	0.18	64	6	0.002	20	5	0.43 *
pos	18	13		9	6		9	7		8	4	
Estrogen receptor												
neg	36	13	0.12									
pos	73	13										
HER2												
neg	95	19	0.13	28	9	0.71 *	67	10	0.11 *			
pos	14	7		8	4		6	3				
p-STAT3												
neg	53	13	1	19	2	0.025 *	34	11	0.015 *	16	1	0.023 *
pos	56	13		17	11		39	2		12	8	

* Fisher’s exact test was used.

**Table 3:** Correlation between p-STAT3 expression and pathological factors in all cases, ER(−) cases, ER(+) cases and TNBC cases.

Variables	All Cases (n = 135)			ER(−) Cases (n = 49)			ER(+) Cases (n = 86)			TNBC (n = 37)		
p-STAT3	Negative	Positive	*p*-Value	Negative	Positive	*p*-Value	Negative	Positive	*p*-Value	Negative	Positive	*p*-Value
Tumor size												
1	31	32	1	10	12	0.78	21	20	1	9	10	1
2,3	35	37		11	16		24	21		8	10	
Lymph node metastasis												
neg	42	41	0.72	13	16	0.78	29	25	0.83	11	12	1
pos	24	28		8	12		16	16		6	8	
Nuclear grade												
1,2	55	54	0.51	15	17	0.55	40	37	1	11	12	1
3	11	15		6	11		5	4		6	8	
Blood vessel invasion												
neg	57	62	0.6	18	27	0.3 *	39	35	1	15	19	0.58 *
pos	9	7		3	1		6	6		2	1	
Lymphatic vessel invasion												
neg	50	54	0.84	16	18	0.53 *	34	36	0.17	13	12	0.32 *
pos	16	15		5	10		11	5		4	8	
Estrogen receptor												
neg	21	28	0.37	21	28		0	0		17	20	
pos	45	41		0	0		45	41		0	0	
HER2												
neg	59	55	0.16	17	20	0.52*	42	35	0.3*	17	20	
pos	7	14		4	8		3	6		0	0	
10-year RFS rate (%)	87.9	82.6		90.5	64.3		95.6	85.4		94.1	65	

* Fisher’s exact test was used.

**Table 4:** Cox proportional hazards regression model analysis of RFS in all cases, ER(−) cases, ER(+) cases and TNBC cases.

Covariate	Categories	All Cases (n = 135)				ER(−) Cases (n = 49)				ER(+) Cases (n = 86)				TNBC (n = 37)			
		Univariate Analysis		Multivariate Analysis		Univariate Analysis		Multivariate Analysis		Univariate Analysis		Multivariate Analysis		Univariate Analysis		Multivariate Analysis	
		HR (95%CI)	*p*-Value	HR (95%CI)	*p*-Value	HR (95%CI)	*p*-Value	HR (95%CI)	*p*-Value	HR (95%CI)	*p*-Value	HR (95%CI)	*p*-Value	HR (95%CI)	*p*-Value	HR (95%CI)	*p*-Value
Tumor size	1 vs. 2,3	3.31 (1.33–8.25)	0.01	1.88 (0.70–5.08)	0.21	12.43 (1.61–95.80)	0.016	13.03 (1.69–100.50)	0.014	1.52 (0.50–4.63)	0.47			10.32 (1.29–82.67)	0.028	11.50 (1.43–92.51)	0.022
Lymph node metastasis	neg vs. pos	4.10 (1.78–9.44)	<0.001	2.31 (0.87–6.09)	0.092	2.53 (0.83–7.75)	0.1			6.53 (1.80–23.73)	0.0044	3.96 (0.87–17.84)	0.073	2.14 (0.57–7.97)	0.26		
Nuclear grade	1,2 vs. 3	1.03 (0.63–1.67)	0.92			0.93 (0.52–1.68)	0.81			0.84 (0.30–2.34)	0.74			0.94 (0.47–1.87)	0.85		
Blood vessel invasion	neg vs. pos	2.60 (1.04–6.49)	0.041			0.96 (0.12–7.39)	0.97			5.01 (1.63–15.37)	0.0048	1.76 (0.50–6.17)	0.38	1.50 (0.19–12.04)	0.7		
Lymphatic vessel invasion	neg vs. pos	4.13 (1.91–8.94)	<0.001	2.23 (0.92–5.40)	0.075	2.12 (0.71–6.31)	0.18			6.97 (2.33–20.80)	<0.001	2.76 (0.81–9.36)	0.1	1.80 (0.48–6.71)	0.38		
Estrogen receptor	neg vs. pos	0.50 (0.23–1.08)	0.079														
HER2	neg vs. pos	2.34 (0.98–5.57)	0.055			1.57 (0.48–5.11)	0.45			2.85 (0.78–10.37)	0.11						
p-STAT3	neg vs. pos	1.00 (0.46–2.15)	0.99			5.05 (1.12–22.83)	0.035	5.37 (1.19–24.29)	0.029	0.18 (0.04–0.81)	0.027	0.18 (0.03–0.82)	0.026	8.41 (1.05–67.32)	0.045	9.48 (1.18–76.21)	0.034

HR: hazard ratio, CI: confidence interval.

## Data Availability

The datasets analyzed in the present study are available from the corresponding author on reasonable request.
